# Correction: Mechanism unravelling for ultrafast and selective ^99^TcO_4_^–^ uptake by a radiation-resistant cationic covalent organic framework: a combined radiological experiment and molecular dynamics simulation study

**DOI:** 10.1039/c9sc90094b

**Published:** 2019-05-02

**Authors:** Linwei He, Shengtang Liu, Long Chen, Xing Dai, Jie Li, Mingxing Zhang, Fuyin Ma, Chao Zhang, Zaixing Yang, Ruhong Zhou, Zhifang Chai, Shuao Wang

**Affiliations:** a State Key Laboratory of Radiation Medicine and Protection , School of Radiation Medicine and Protection , Collaborative Innovation Center of Radiological Medicine of Jiangsu Higher Education Institutions , Soochow University , Suzhou 215123 , China . Email: shuawang@suda.edu.cn; b Shanghai Institute of Applied Physics , Chinese Academy of Sciences , No. 2019 Jialuo Rd., Jiading Dist. , Shanghai , 201800 , China; c School of Materials Science and Engineering , Anhui University of Science and Technology , Huainan 232001 , China

## Abstract

Correction for ‘Mechanism unravelling for ultrafast and selective ^99^TcO_4_^–^ uptake by a radiation-resistant cationic covalent organic framework: a combined radiological experiment and molecular dynamics simulation study’ by Linwei He *et al.*, *Chem. Sci.*, 2019, DOI: ; 10.1039/c9sc00172g.



## 

An error occurred when we plotted some of the figures in this paper, caused by totally unintentional misoperation. When plotting Fig. 2d, e and S5, the misoperation of batch input of the XRD data leads to the repeating patterns in the above mentioned figures. We have rechecked our raw data carefully and would like to make a correction to Fig. 2d and e using the updated figures generated by the raw data shown below. An updated ESI file containing the correct version of Fig. S5 is also provided.

**Fig. 1:**
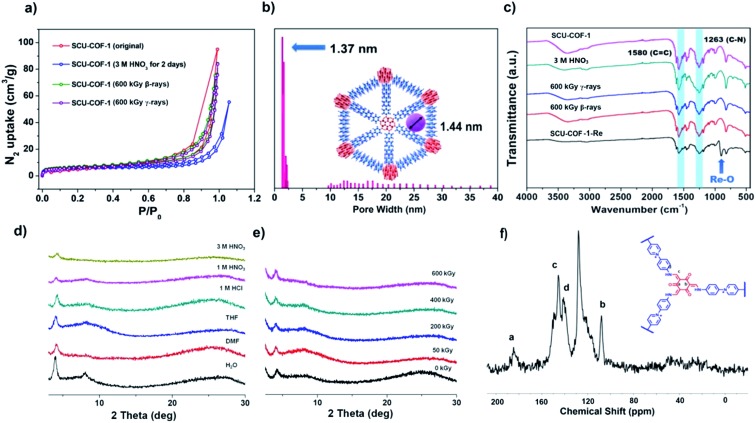
(a) N_2_ sorption isotherms of SCU-COF-1 after different treatments with 3 M HNO_3_, 600 kGy β-rays, and 600 kGy γ-rays at 77 K. (b) Pore-size distribution of SCU-COF-1 calculated by NLDFT modelling based on N_2_ adsorption data. (c) IR spectra of SCU-COF-1 and SCU-COF-1 after treatment with 3 M HNO_3_, 600 kGy γ-irradiation, 600 kGy β-irradiation, and stock solution containing ReO_4_^–^. (d) PXRD patterns of SCU-COF-1 after treatments in various solvents and acids for 48 h. (e) PXRD patterns of SCU-COF-1 after being irradiated with various doses of β-irradiation (50 kGy, 200 kGy, 400 kGy, 600 kGy). (f) ^13^C solid NMR spectrum of SCU-COF-1.

The Royal Society of Chemistry apologises for these errors and any consequent inconvenience to authors and readers.

